# Prognostic role of SIRT6 in gastrointestinal cancers: a meta-analysis

**DOI:** 10.1515/med-2020-0403

**Published:** 2020-04-21

**Authors:** Li Shi, Ying Wang, Timothy Bonney Oppong, Xiaoli Fu, Haiyan Yang, Yadong Wang

**Affiliations:** Department of Epidemiology, School of Public Health, Zhengzhou University, Zhengzhou 450001, China; Department of Social Medicine and Health Management, School of Public Health, Zhengzhou University, Zhengzhou 450001, China; Department of Toxicology, Henan Center for Disease Control and Prevention, Zhengzhou 450016, China

**Keywords:** SIRT6, gastrointestinal cancers, overall survival, prognosis, meta-analysis

## Abstract

Sirtuin 6 (SIRT6) plays a critical role in the progression and development of gastrointestinal cancers. However, the association between SIRT6 expression and clinicopathological parameters and prognosis in gastrointestinal cancer patients remains inconclusive. Consequently, we conducted this meta-analysis to evaluate the importance of SIRT6 expression in various types of gastrointestinal cancers. PubMed, EMBASE, and Web of Science databases were systematically searched to screen the relevant literature. The reported or estimated hazard ratio (HR) and odds ratio (OR) and their corresponding 95% confidence interval (CI) were pooled to assess the strength of the association. Nine studies involving 867 patients were included in the meta-analysis. Overall analysis showed that high SIRT6 expression was related to better overall survival in gastrointestinal cancers (HR = 0.62, 95% CI = 0.47–0.82). High SIRT6 expression was also related to a favorable tumor node metastasis (TNM) stage (OR = 0.44, 95% CI = 0.28–0.70) among gastrointestinal cancer patients. Our meta-analysis revealed that high SIRT6 expression might be a potential biomarker predicting better prognosis in gastrointestinal cancers, which may offer options for gastrointestinal cancer treatment.

## 1 Introduction

Cancers of the digestive system are one of the most common types in aggressive malignancies worldwide. In 2019, almost 3,28,030 new cancer cases and 1,65,460 cancer deaths of the digestive system were assumed to occur in the United States according to the International Agency for Research on Cancer [1]. The development of therapeutic methods including surgery, radiotherapy, and chemotherapy is of potential benefit to gastrointestinal cancer patients; however, the prognosis and overall survival (OS) of gastrointestinal cancer patients remain poor. Thus, it is of great significance to discover a promising biomarker to improve the prognosis and quality of life of gastrointestinal cancer patients.

Epigenetic alterations including DNA methylation, chromatin remodeling, histone modifications, and noncoding RNAs are a hallmark of gastrointestinal cancers [2]. Among these, histone acetylation has become a research hotspot in recent years. Sirtuins (SIRTs) are class III histone deacetylases and SIRTs using NAD^+^ as a co-substrate for their enzymatic activities have seven different members (SIRT1–SIRT7) in mammals [3]. SIRT1, SIRT6, and SIRT7 are localized in the nucleus. SIRT2 is cytoplasmic, and SIRT3, SIRT4, and SIRT5 are mitochondrial [4]. SIRT1, the most extensively studied member of mammalian SIRTs, deacetylates not only histone but also nonhistone proteins to regulate many biological processes, such as cell stress response, apoptosis, senescence, and DNA repair [5]. In addition, SIRT1 is regarded as a prognostic marker for OS in gastrointestinal cancers [6]. In addition, SIRT6 and SIRT1 have similar functions, and SIRT6 modulates various physiological processes, including aging, metabolism, telomere maintenance, and genomic DNA stability and repair [7,8,9]. Recently, the role of SIRT6 in tumor development has been partly studied. SIRT6 expression was abnormal in various gastrointestinal tumor tissues, including colorectal cancer [10,11,12,13,14], gastric cancer (GC) [15], pancreatic cancer [16,17], and hepatocellular carcinoma (HCC) [18,19]. However, the prognostic role of SIRT6 in gastrointestinal cancers remains inconsistent and controversial according to the available evidence [10,11,12,15,17,18]. Hence, it is necessary to perform this meta-analysis to systematically evaluate the relationship between SIRT6 expression and OS and clinicopathological parameters in gastrointestinal cancers through the collection of published evidence.

## 2 Materials and methods

### 2.1 Search strategy

Published literature on SIRT6 and gastrointestinal cancer was systematically searched in PubMed, EMBASE, and Web of Science databases by two independent authors (up to March 2020). The following terms were used for the search: “sirtuin 6” OR “SIRT6” AND “cancer” OR “tumor” OR “carcinoma”. Furthermore, “cancer” was replaced by the name of each gastrointestinal cancer (such as GC) to identify any missed papers. In addition, references were also screened in case articles were missed. The search was done with no limitation on country and race.

### 2.2 Inclusion and exclusion criteria

The included studies had to meet the following criteria: (1) the published papers were in English; (2) explore the prognostic role of SIRT6 in gastrointestinal cancer patients; (3) group the SIRT6 high or positive expression and SIRT6 low or negative expression; (4) provide the association between clinicopathological features and SIRT6 expression; (5) contain the relationship between SIRT6 expression and OS in gastrointestinal cancer patients; and (6) report the sufficient data to obtain the hazard ratio (HR). The exclusion criteria involve (1) studies with unusable or insufficient data; (2) meeting abstracts, reviews, or letters; (3) animal or cell studies; and (4) overlapping publications.

### 2.3 Data extraction and quality assessment

The following data from eligible studies were respectively collected by two authors: author name, publication year, patient source, tumor type, total number, method, and clinicopathological parameters such as HR and corresponding 95% confidence interval (CI) for OS. Multivariate analysis was considered if both multivariate analysis and univariate analysis were provided. The Newcastle–Ottawa scale score was calculated to assess the quality of the studies. The total scores ranged from 0 to 9, and a score of 6 or more was deemed to be a high-quality study [20].

### 2.4 Statistical methods

The relationship between the expression of SIRT6 and OS in gastrointestinal cancer patients was assessed by using HR and 95% CI, but the odds ratio (OR) and 95% CI were utilized to evaluate the association between SIRT6 expression and clinical parameters. The HR and 95% CI for OS were estimated by the Kaplan–Meier curve with the help of the Engauge Digitizer 10.8 software [21]. The heterogeneity test was performed by *I* ^2^ test (*I* ^2^ < 50% for the fixed-effects model; *I* ^2^ ≥ 50% for the random-effects model) [22]. Sensitivity analysis was conducted to assess the robustness and reliability of the results. The publication bias was evaluated through Begg’s test and Egger’s test. STATA 11.2 software was utilized to perform all statistical analyses. Two-tailed *P* < 0.05 was regarded as statistically significant.

## 3 Results

### 3.1 Study characteristics

**Figure 1:**
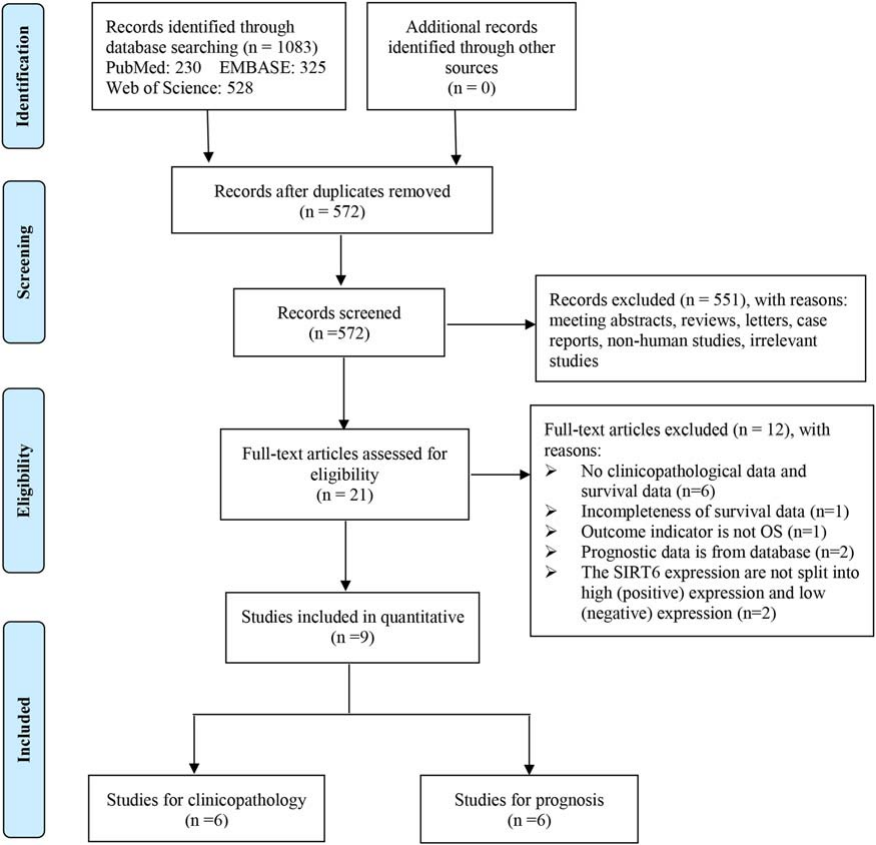
Flow diagram of study search and selection process.

A total of 1,083 records were identified from PubMed (*n* = 230), EMBASE (*n* = 325), and Web of Science (*n* = 528); 511 studies were deemed duplicate publications and thus removed, and 551 were excluded based on their titles and abstracts. Thereafter, 12 of the remaining 21 studies were excluded during full-text review because they did not meet the inclusion criteria. Finally, a total of nine studies with 867 cases (Figure 1) met the inclusion criteria and thus were included in this study. Among these studies, five explored colorectal cancer (CRC) and one each explored esophageal squamous cell carcinoma, GC, pancreatic ductal adenocarcinoma (PDAC), and HCC. The characteristics of these included studies are indicated in Table 1.

**Table 1:** Characteristics of the studies included in this meta-analysis

Study	Year	Patient source	Tumor type	Total number	Method	Outcome	Clinicopathological factors	NOS score
Zhang et al. [10]	2019	China	CRC	50	IHC	OS	NA	6
Geng et al. [13]	2018	China	CRC	196	IHC	NA	Tumor size, LNM, differentiation	6
Li et al. [12]	2018	China	CRC	97	IHC	OS	TNM	8
Qi et al. [14]	2018	China	CRC	113	IHC	NA	Tumor size, LNM, differentiation, distant metastasis, TNM	7
Tian and Yuan [11]	2018	China	CRC	90	IHC	OS	TNM	6
Huang et al. [23]	2017	China	ESCC	80	IHC	NA	LNM, differentiation, TNM	5
Zhou et al. [15]	2017	China	GC	68	IHC	OS	Distant metastasis, TNM	6
Kugel et al. [17]	2016	USA	PDAC	120	IHC	OS	NA	6
Ran et al. [18]	2016	China	HCC	53	WB	OS	NA	6

Abbreviations: CRC, colorectal cancer; ESCC, esophageal squamous cell carcinoma; GC, gastric cancer; PDAC, pancreatic ductal adenocarcinoma; HCC, hepatocellular carcinoma; OS, overall survival; IHC, immunohistochemistry; WB: western blotting; LNM: lymph node metastasis; TNM: tumor node metastasis; NA, not applicable; and NOS, Newcastle–Ottawa scale.

### 3.2 Relationship between SIRT6 expression and OS

**Figure 2:**
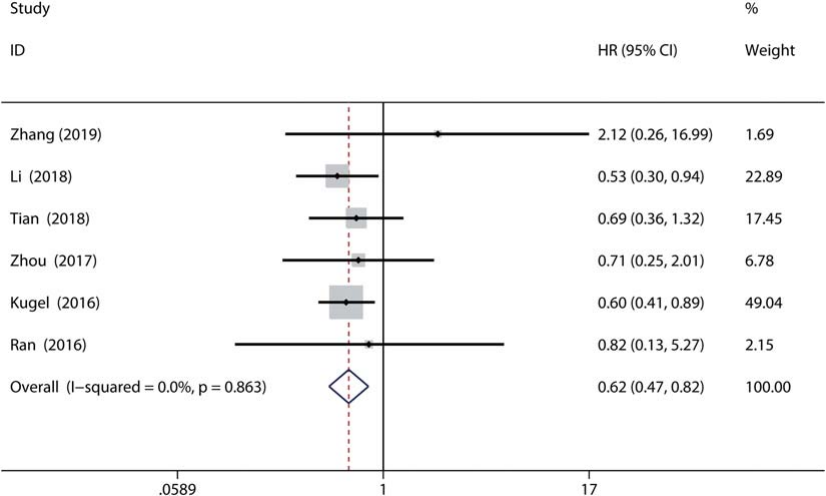
Forest plot for the association between SIRT6 expression and OS in gastrointestinal cancers. Solid diamonds: the HR of each study; squares: weight of each study; horizontal line: the 95% CI of each study; dotted line: the pooled HR; and unfilled diamond: the pooled results for all studies.

Six of the included studies with 501 patients were used to investigate the relationship between high SIRT6 expression and OS in gastrointestinal cancers [10,11,12,15,17,18]. The pooled HR for OS showed that high SIRT6 expression was related to better OS in gastrointestinal cancers (HR = 0.62, 95% CI = 0.47–0.82, *P* = 0.001; *I* ^2^ = 0.0%, *P* = 0.863, fixed-effects model) (Figure 2).

### 3.3 Relationship between SIRT6 expression and clinicopathological features

**Figure 3:**
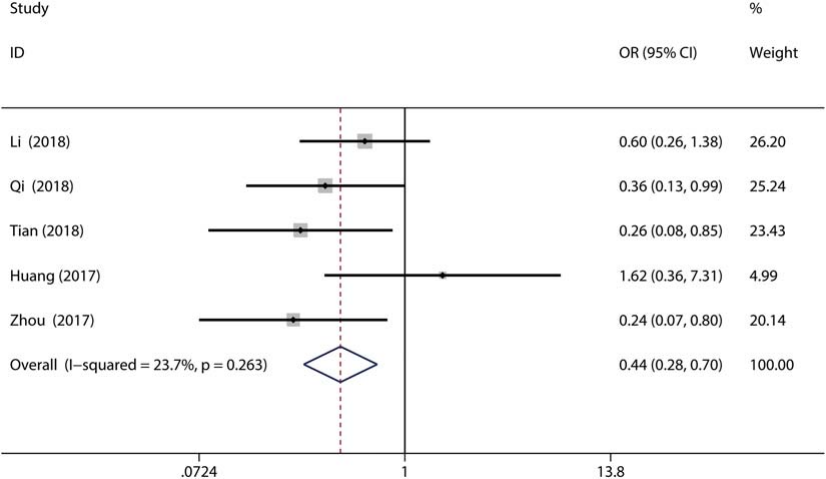
Forest plot for the association between SIRT6 expression and TNM stage in gastrointestinal cancers. Solid diamonds: the OR of each study; squares: weight of each study; horizontal line: the 95% CI of each study; dotted line: the pooled odd ratio; and unfilled diamond: the pooled results for all studies.

Six of the included studies with 644 patients were used to analyze the relationship between high SIRT6 expression and clinical parameters [11,12,13,14,15,23]. The results showed that high SIRT6 expression was related to a favorable tumor node metastasis (TNM) stage (OR = 0.44, 95% CI = 0.28–0.70, *P* = 0.001; *I* ^2^ = 23.7%, *P* = 0.263, fixed-effects model) (Figure 3 and Table 2). However, there was no significant association between SIRT6 expression and tumor size, differentiation, distant metastasis, or lymph node metastasis (Table 2).

**Table 2:** The relationship between SIRT6 expression and clinicopathological characteristics

Characteristics	Studies	Case number	Pooled OR (95% CI)	*P* value	Heterogeneity		Model	Begg’s test (*P* value)	Egger’s test (*P* value)
					*I* ^2^ (%)	*P*			
Tumor size (>3 cm vs ≤3 cm)	2	309	1.12 (0.30–4.24)	0.865	83.9	0.013	Random	1	—
Lymph node metastasis (yes vs no)	3	389	0.75 (0.12–4.57)	0.752	88.6	<0.001	Random	1	0.265
Differentiation (well/moderate vs poor)	3	386	0.94 (0.56–1.58)	0.806	0.0	0.966	Fixed	1	0.764
Distant metastasis (yes vs no)	2	181	1.19 (0.14–9.93)	0.872	78	0.033	Random	1	—
Tumor node metastasis (III + IV vs I + II)	5	443	0.44 (0.28–0.70)	0.001	23.7	0.263	Fixed	0.806	0.846

### 3.4 Sensitivity analysis and publication bias

**Figure 4:**
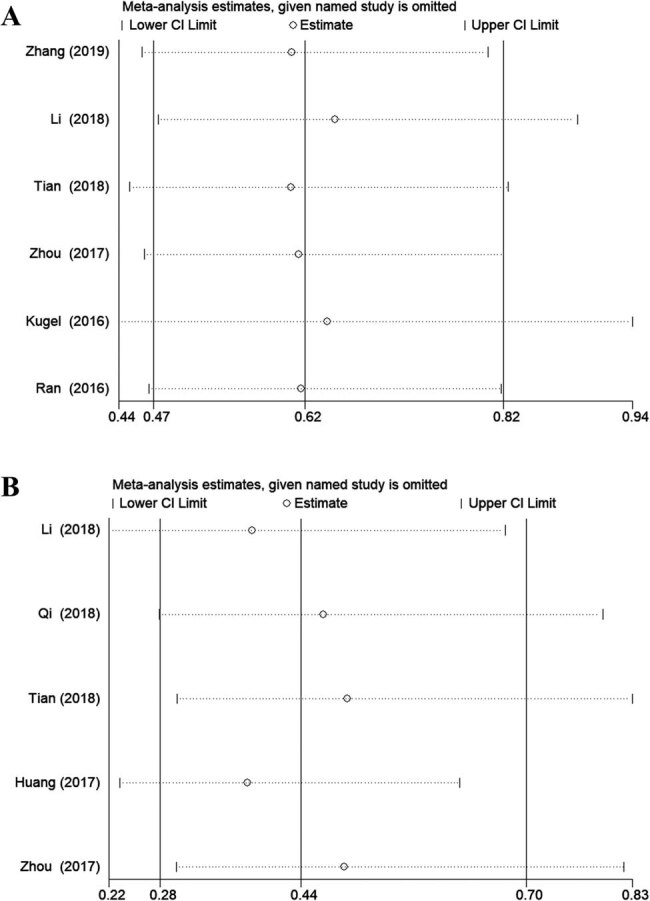
Sensitivity analyses of the studies: (A) OS and (B) TNM stage. Unfilled circles: the ratio of each study; horizontal dotted line: the 95% CI of each study; solid line on the left: the lower 95% CI of the pooled results; solid line on the right: the upper 95% CI of the pooled results; and solid line in the middle: the pooled ratios of all studies.

**Figure 5:**
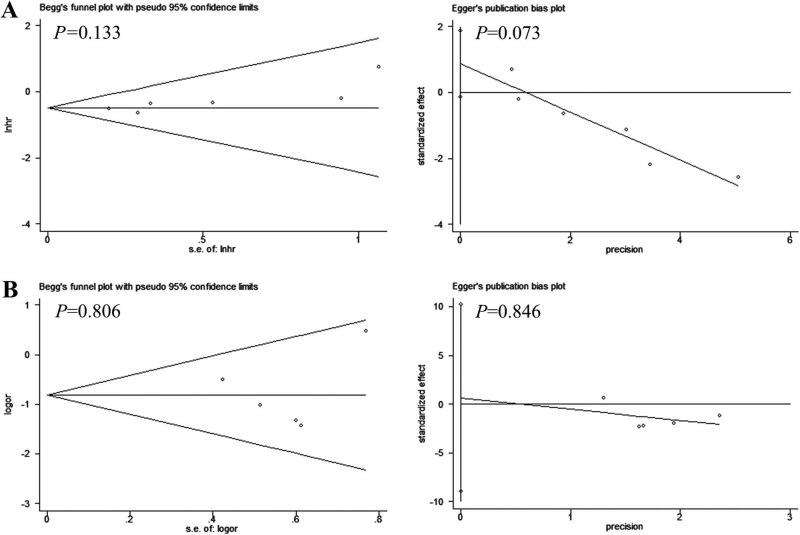
Begg’s funnel plot and Egger’s test for publication bias. (A) Overall survival and (B) TNM stage.

Sensitivity analysis was conducted to evaluate the robustness and reliability of the results. The sensitivity analysis indicated that our results were stable (Figure 4). Begg’s test and Egger’s test were used to assess the publication bias. Both tests (OS: *P* = 0.133; TNM: *P* = 0.806 and OS: *P* = 0.073; TNM: *P* = 0.846, respectively) showed that no publication bias existed in any analyses (Figure 5).

## 4 Discussion

SIRT6 is a nuclear protein possessing deacetylase and ADP-ribosyltransferase activity [24]. SIRT6 is closely related to chromatin, deacetylates H3K9 and H3K56, and regulates glucose metabolism, inflammation, gene expression, and genomic stability [25,26,27,28,29,30], which is associated with tumor survival. In this study, we performed a meta-analysis of nine studies including 867 cancer patients and found that high expression of SIRT6 was significantly related to longer OS time in gastrointestinal cancers including CRC, GC, PDAC, and HCC. In addition, we explored the relationship between SIRT6 expression and TNM stage. We found that more positive sections were detected in stage I/II patients compared with stage III/IV patients among gastrointestinal cancers, which suggested that high SIRT6 expression was related to favorable gastrointestinal cancer features.

There are several underlying mechanisms involved in the tumor suppressor role of SIRT6. In human colon cancer, USP10 and SIRT6 protein expressions were reduced, and USP10 antagonized c-Myc transcriptional activity through SIRT6 and p53 to inhibit cell cycle progression, cancer cell growth, and tumor formation [31]. SIRT6 suppressed HCC cell growth via inhibition of the extracellular signal-regulated kinase signaling pathway [32]. SIRT6 suppressed pancreatic cancer through the control of Lin-28b [17]. Liu et al. showed that knockdown of SIRT6 promoted invasion of hepatoma HepG2 and Huh7 cells *in vitro* [33]. Tian and Yuan reported that overexpression of SIRT6 inhibited migration and invasion in colon cancer cells *in vitro* [11]. Bhardwaj and Das indicated that the ectopic expression of SIRT6 inhibited the migratory and invasive ability of hepatoma HepG2 cells *in vitro* [34]. These studies argued for a cancer-inhibiting function of SIRT6 in these cancers.

TNM stage is mainly influenced by proliferation and apoptosis. Previously published studies in animals and human cells supported the results of our study. SIRT6 deficiency could increase the incidence of invasive colonic adenocarcinoma in a mouse model expressing an adenomatosis polyposis coli mutation [35]. Overexpression of SIRT6 induced apoptosis in HCC cells and could also reduce tumor formation and tumor growth of HCC cells in *in vitro* and *in vivo* experiments [19,32]. Furthermore, SIRT6 affected cancer cell proliferation by suppressing the transcriptional activity of c-Myc [31].

The potential role of epigenetic biomarkers in prognosis is crucial for the treatment of gastrointestinal cancers. Recently, the findings from emerging studies have indicated that several prognostic biomarkers including BCL6B, CDKN2A, and BORIS may play a significant role in gastrointestinal cancer treatment. However, few of them have been used in the clinical applications [2]. Regarding SIRT6, it is involved in regulating the energy metabolism of tumors as a tumor suppressor. Zhong et al. indicated that SIRT6-deficient cells revealed increased HIF1α activity and glucose uptake [28]. In addition, SIRT6 could kill cancer cells. Zhang and Qin showed that knockdown of SIRT6 promoted growth of the HepG2 cells, while SIRT6 overexpression inhibited its growth [32]. Also, SIRT6 could inhibit signaling pathways associated with tumorigenesis. Min et al. reported that c-Fos induced SIRT6 transcription inhibiting survivin through the reduction of histone H3K9 acetylation and NF-κB activation [36]. Finally, SIRT6 could increase the sensitivity to tumor treatment. Marquardt et al. exhibited that SIRT6 overexpression in HepG2 cells increased apoptosis sensitivity to CD95 stimulation or chemotherapy treatment [37]. Although the role of SIRT6 in tumorigenesis and development still have many unanswered questions, SIRT6 would be utilized for cancer prevention and site-specific treatment, especially for cancer nanomedicine [38].

To our knowledge, our study was the first meta-analysis to assess the clinical value of the SIRT6 expression level in gastrointestinal cancer patients. However, there are some limitations in this study. First, we could not adequately analyze the association between SIRT6 expression and each gastrointestinal cancer type and specific clinical parameters because of the limited publications and the lack of significant data. Second, we had to estimate HR and 95% CI for OS by the Kaplan–Meier curve when we could not directly extract data from the study. Again, the method and cut-off value grouping high or low expression of SIRT6 varied among these studies, which may result in potential bias. Finally, the populations of most of the included studies are in China, and the results acquired may be carefully generalizable outside this population. More studies and larger sample sizes are needed to resolve these limitations.

In conclusion, our findings demonstrated that high SIRT6 expression was associated with longer OS time in gastrointestinal cancers and favorable TNM stage. More large-scale and well-matched studies are warranted to identify the role of SIRT6 in different gastrointestinal cancer type prognoses and clinical applications.

## Abbreviations

CRC: colorectal cancer
CI: confidence interval
ESCC: esophageal squamous cell carcinoma
GC: gastric cancer
HCC: hepatocellular carcinoma
HR: hazard ratio
IHC: immunohistochemistry
LNM: lymph node metastasis
NOS: Newcastle–Ottawa scale
NA: not applicable
OS: overall survival
OR: odds ratio
PDAC: pancreatic ductal adenocarcinoma
SIRT6: sirtuin 6
TNM: tumor node metastasis
WB: western blotting

## References

[1] Siegel RL, Miller KD, Jemal A. Cancer statistics, 2019. CA Cancer J Clin. 2019;69(1):7–34. 10.3322/caac.21551. 30620402

[2] Wong CC, Li W, Chan B, Yu J. Epigenomic biomarkers for prognostication and diagnosis of gastrointestinal cancers. Semin Cancer Biol. 2019;55:90–105. 10.1016/j.semcancer.2018.04.002. 29665409

[3] Arrowsmith CH, Bountra C, Fish PV, Lee K, Schapira M. Epigenetic protein families: a new frontier for drug discovery. Nat Rev Drug Discovery. 2012;11(5):384–400. 10.1038/nrd3674. 22498752

[4] Kida Y, Goligorsky MS. Sirtuins, cell senescence, and vascular aging. Can J Cardiol. 2016;32(5):634–41. 10.1016/j.cjca.2015.11.022. PMC4848124 26948035

[5] Chen Y, Wang T, Wang W, Hu J, Li R, He S, et al. Prognostic and clinicopathological significance of SIRT1 expression in NSCLC: a meta-analysis. Oncotarget. 2017;8(37):62537–44. 10.18632/oncotarget.19244. PMC5617527 28977967

[6] Wu S, Jiang J, Liu J, Wang X, Gan Y, Tang Y. Meta-analysis of SIRT1 expression as a prognostic marker for overall survival in gastrointestinal cancer. Oncotarget. 2017;8(37):62589–99. 10.18632/oncotarget.19880. PMC5617531 28977971

[7] Houtkooper RH, Pirinen E, Auwerx J. Sirtuins as regulators of metabolism and healthspan. Nat Rev Mol Cell Biol. 2012;13(4):225–38. 10.1038/nrm3293. PMC4872805 22395773

[8] Chalkiadaki A, Guarente L. The multifaceted functions of sirtuins in cancer. Nat Rev Cancer. 2015;15(10):608–24. 10.1038/nrc3985. 26383140

[9] Jia G, Su L, Singhal S, Liu X. Emerging roles of SIRT6 on telomere maintenance, DNA repair, metabolism and mammalian aging. Mol Cell Biochem. 2012;364(1–2):345–50. 10.1007/s11010-012-1236-8. 22286818

[10] Zhang Y, Nie L, Xu K, Fu Y, Zhong J, Gu K, et al. SIRT6, a novel direct transcriptional target of FoxO3a, mediates colon cancer therapy. Theranostics. 2019;9(8):2380–94. 10.7150/thno.29724. PMC6531295 31149050

[11] Tian J, Yuan L. Sirtuin 6 inhibits colon cancer progression by modulating PTEN/AKT signaling. Biomed Pharmacother. 2018;106:109–16. 10.1016/j.biopha.2018.06.070. 29957460

[12] Li N, Mao D, Cao Y, Li H, Ren F, Li K. Downregulation of SIRT6 by miR-34c-5p is associated with poor prognosis and promotes colon cancer proliferation through inhibiting apoptosis via the JAK2/STAT3 signaling pathway. Int J Oncol. 2018. 10.3892/ijo.2018.4304. PMC5873872 29512698

[13] Geng CH, Zhang CL, Zhang JY, Gao P, He M, Li YL. Overexpression of Sirt6 is a novel biomarker of malignant human colon carcinoma. J Cell Biochem. 2018;119(5):3957–67. 10.1002/jcb.26539. 29227545

[14] Qi J, Cui C, Deng Q, Wang L, Chen R, Zhai D, et al. Downregulated SIRT6 and upregulated NMNAT2 are associated with the presence, depth and stage of colorectal cancer. Oncol Lett. 2018;16(5):5829–37. 10.3892/ol.2018.9400. PMC6176414 30333863

[15] Zhou J, Wu A, Yu X, Zhu J, Dai H. SIRT6 inhibits growth of gastric cancer by inhibiting JAK2/STAT3 pathway. Oncol Rep. 2017;38(2):1059–66. 10.3892/or.2017.5753. 28656307

[16] Bauer I, Grozio A, Lasiglie D, Basile G, Sturla L, Magnone M, et al. The NAD+-dependent histone deacetylase SIRT6 promotes cytokine production and migration in pancreatic cancer cells by regulating Ca2+ responses. J Biol Chem. 2012;287(49):40924–37. 10.1074/jbc.M112.405837. PMC3510797 23086953

[17] Kugel S, Sebastian C, Fitamant J, Ross KN, Saha SK, Jain E, et al. SIRT6 suppresses pancreatic cancer through control of Lin28b. Cell. 2016;165(6):1401–15. 10.1016/j.cell.2016.04.033. PMC4892983 27180906

[18] Ran LK, Chen Y, Zhang ZZ, Tao NN, Ren JH, Zhou L, et al. SIRT6 overexpression potentiates apoptosis evasion in hepatocellular carcinoma via BCL2-associated X protein-dependent apoptotic pathway. Clin Cancer Res. 2016;22(13):3372–82. 10.1158/1078-0432.CCR-15-1638. 26861461

[19] Wang Y, Pan T, Wang H, Li L, Li J, Zhang D, et al. Overexpression of SIRT6 attenuates the tumorigenicity of hepatocellular carcinoma cells. Oncotarget. 2017;8(44):76223–30. 10.18632/oncotarget.19297. PMC5652700 29100306

[20] Stang A. Critical evaluation of the Newcastle–Ottawa scale for the assessment of the quality of nonrandomized studies in meta-analyses. Eur J Epidemiol. 2010;25(9):603–5. 10.1007/s10654-010-9491-z. 20652370

[21] Tierney JF, Stewart LA, Ghersi D, Burdett S, Sydes MR. Practical methods for incorporating summary time-to-event data into meta-analysis. Trials. 2007;8:16. 10.1186/1745-6215-8-16. PMC1920534 17555582

[22] DerSimonian R, Laird N. Meta-analysis in clinical trials revisited. Contemp Clin Trials. 2015;45(Pt A):139–45. 10.1016/j.cct.2015.09.002. PMC4639420 26343745

[23] Huang N, Liu Z, Zhu J, Cui Z, Li Y, Yu Y, et al. Sirtuin 6 plays an oncogenic role and induces cell autophagy in esophageal cancer cells. Tumour Biol. 2017;39(6):1010428317708532. 10.1177/1010428317708532. 28653878

[24] Polyakova O, Borman S, Grimley R, Vamathevan J, Hayes B, Solari R. Identification of novel interacting partners of Sirtuin6. PLoS One. 2012;7(12):e51555. 10.1371/journal.pone.0051555. PMC3519869 23240041

[25] Michishita E, McCord RA, Berber E, Kioi M, Padilla-Nash H, Damian M, et al. SIRT6 is a histone H3 lysine 9 deacetylase that modulates telomeric chromatin. Nature. 2008;452(7186):492–6. 10.1038/nature06736. PMC2646112 18337721

[26] Michishita E, McCord RA, Boxer LD, Barber MF, Hong T, Gozani O, et al. Cell cycle-dependent deacetylation of telomeric histone H3 lysine K56 by human SIRT6. Cell Cycle. 2009;8(16):2664–6. 10.4161/cc.8.16.9367. PMC4474138 19625767

[27] Kugel S, Mostoslavsky R. Chromatin and beyond: the multitasking roles for SIRT6. Trends Biochem Sci. 2014;39(2):72–81. 10.1016/j.tibs.2013.12.002. PMC3912268 24438746

[28] Zhong L, D’Urso A, Toiber D, Sebastian C, Henry RE, Vadysirisack DD, et al. The histone deacetylase Sirt6 regulates glucose homeostasis via Hif1alpha. Cell. 2010;140(2):280–93. 10.1016/j.cell.2009.12.041. PMC2821045 20141841

[29] Mostoslavsky R, Chua KF, Lombard DB, Pang WW, Fischer MR, Gellon L, et al. Genomic instability and aging-like phenotype in the absence of mammalian SIRT6. Cell. 2006;124(2):315–29. 10.1016/j.cell.2005.11.044. 16439206

[30] Yang B, Zwaans BM, Eckersdorff M, Lombard DB. The sirtuin SIRT6 deacetylates H3 K56Ac in vivo to promote genomic stability. Cell Cycle. 2009;8(16):2662–3. 10.4161/cc.8.16.9329. PMC2728171 19597350

[31] Lin Z, Yang H, Tan C, Li J, Liu Z, Quan Q, et al. USP10 antagonizes c-Myc transcriptional activation through SIRT6 stabilization to suppress tumor formation. Cell Rep. 2013;5(6):1639–49. 10.1016/j.celrep.2013.11.029. PMC4007576 24332849

[32] Zhang ZG, Qin CY. Sirt6 suppresses hepatocellular carcinoma cell growth via inhibiting the extracellular signal regulated kinase signaling pathway. Mol Med Rep. 2014;9(3):882–8. 10.3892/mmr.2013.1879. 24366394

[33] Liu J, Yu Z, Xiao Y, Meng Q, Wang Y, Chang W. Coordination of FOXA2 and SIRT6 suppresses the hepatocellular carcinoma progression through ZEB2 inhibition. Cancer Manag Res. 2018;10:391–402. 10.2147/CMAR.S150552. PMC5836661 29535552

[34] Bhardwaj A, Das S. SIRT6 deacetylates PKM2 to suppress its nuclear localization and oncogenic functions. Proc Natl Acad Sci U S A. 2016;113(5):E538–47. 10.1073/pnas.1520045113. PMC4747762 26787900

[35] Sebastian C, Zwaans BM, Silberman DM, Gymrek M, Goren A, Zhong L, et al. The histone deacetylase SIRT6 is a tumor suppressor that controls cancer metabolism. Cell. 2012;151(6):1185–99. 10.1016/j.cell.2012.10.047. PMC3526953 23217706

[36] Min L, Ji Y, Bakiri L, Qiu Z, Cen J, Chen X, et al. Liver cancer initiation is controlled by AP-1 through SIRT6-dependent inhibition of surviving. Nat Cell Biol. 2012;14(11):1203–11. 10.1038/ncb2590. 23041974

[37] Marquardt JU, Fischer K, Baus K, Kashyap A, Ma S, Krupp M, et al. Sirtuin-6-dependent genetic and epigenetic alterations are associated with poor clinical outcome in hepatocellular carcinoma patients. Hepatology. 2013;58(3):1054–64. 10.1002/hep.26413. PMC3759627 23526469

[38] de Ceu Teixeira M, Sanchez-Lopez E, Espina M, Garcia ML, Durazzo A, Lucarini M, et al. Sirtuins and SIRT6 in Carcinogenesis and in Diet. Int J Mol Sci. 2019;20(19):4945. 10.3390/ijms20194945. PMC6801518 31591350

